# Supplementary material for: WEE1 Inhibitor Adavosertib Exerts Antitumor Effects on Colorectal Cancer, Especially in Cases with p53 Mutations
Source: Cancers (Basel). 2024 Sep 12;16(18):3136. doi: 10.3390/cancers16183136 (PMC11429655; doi:10.3390/cancers16183136)
Supplement: Supplementary file 1 [file cancers-16-03136-s001.zip › cancers-3175715-supplementary.pdf]

Supplementary figure:
WEE1 Inhibitor Adavosertib Exerts Antitumor Effects on Colorectal Cancer, Especially in Cases with *p53* Mutations

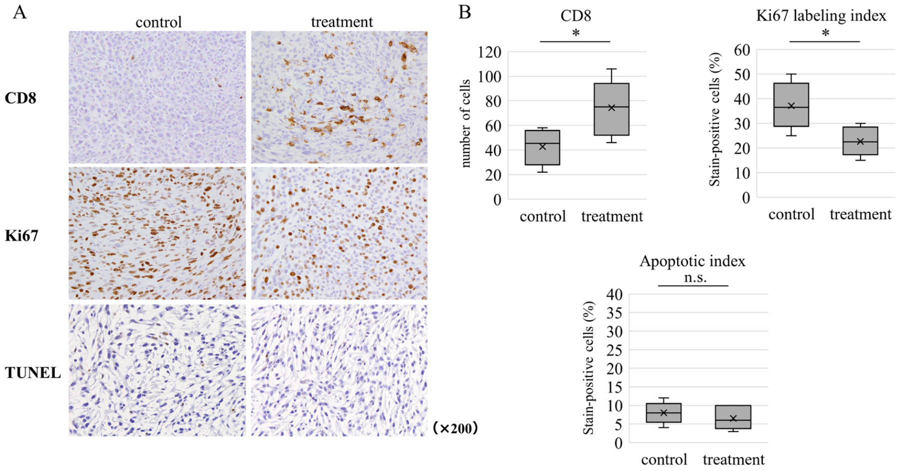

Figure S1. Evaluation of transplanted tumor specimens from a mouse model of CRC created using CT26 by immunohistochemical staining. (A) CD8 and Ki-67 immunostaining and TUNEL staining (200× magnification). (B) Comparison of the number of CD8-positive cells, Ki-67 labeling index, and the percentage of TUNEL staining-positive cells between the control and treatment groups. Welch's *t*-test, *: $p < 0.05$, n.s.: not significant.
